# Experimental Single-Session Imagery Rescripting of Distressing Memories in Bowel/Bladder-Control Anxiety: A Case Series

**DOI:** 10.3389/fpsyt.2014.00182

**Published:** 2014-12-15

**Authors:** Rosanna Pajak, Sunjeev K. Kamboj

**Affiliations:** ^1^Research Department of Clinical, Educational and Health Psychology, University College London, London, UK

**Keywords:** mental imagery, memory, imagery rescripting, bowel-control anxiety, bladder-control anxiety

## Abstract

Bowel and bladder obsession [bowel/bladder-control anxiety (BBCA)] is a viscerally centered phobic syndrome involving a specific concern about losing control of bowel or bladder functioning in a public place. Like other anxiety disorders, BBCA is characterized by intrusive imagery. We have previously described the nature of intrusive mental imagery in BBCA and found imagery themes to be linked to actual experiences of loss of control or to “near misses.” A causal role for imagery in symptom maintenance can be inferred by examining the effects of imagery rescripting. Moreover, successful rescripting may point to a potentially efficacious avenue for treatment development. Three cases of imagery rescripting are described here with pre-, post-, and follow-up (1-week) data reported. After rescripting, two participants experienced pronounced reductions in imagery vividness, distress, shame, disgust, and belief conviction. Most importantly, all three participants experienced a reduction in fear-associated bladder and/or bowel sensations. The results support a causal role for mental imagery in bowel-bladder-control anxiety and suggest that rescripting of distressing intrusive memories linked to recurrent images may be a useful avenue for development of cognitive-behavioral treatments of bladder/bowel-control anxiety.

## Introduction

Acute anxiety is commonly accompanied by an urge to urinate or defecate, reflecting changes in autonomic functioning (1). In some individuals, these visceral sensations are exaggerated and become the focus of catastrophic fears accompanied by the belief that they signal the imminent loss of control of bowel or bladder functioning. This phenomenon has been referred to as bowel- and bladder-*obsession* (2–6), reflecting the tendency to repeatedly check for internal sensation and compulsive voiding behavior. In our recent study, we referred to this syndrome as bowel/bladder-control anxiety (BBCA) (7) and highlighted the additional, perhaps more pronounced overlap with panic.

Bowel/bladder-control anxiety is typified by an overwhelming fear of loss of control of bowel or bladder functioning as well as a variety of sensory and behavioral signs. In addition, catastrophic mental imagery also often accompanies BBCA (8). In anxiety disorders, catastrophic imagery is generally a distorted representation of past or future events and is thought to play a key role in disorder maintenance, while being maintained, in turn, through behavioral and cognitive avoidance (9).

In non-treatment seeking individuals with BBCA (7), the prevalence of intrusive mental imagery was found to be strikingly high (70%), compared to patient groups with anxiety disorders (10). A study of the imagery associated with BBCA (8) found that intrusive and distressing mental images were predominantly visual and accompanied by intense physical (visceral) sensations. The majority of mental imagery (85%) was future-oriented, ending with (or just before) the feared catastrophe (public incontinence) occurs. Importantly, distressing past events significantly influenced the content of participants’ imagery. Sensory fragments of memories (of events related to loss of control) were often superimposed onto images of contemporary anxiety-provoking situations (7). In BBCA, the fragmentary nature of memory-based intrusive images can be understood in the context of high levels of shame, fear, and intense physical sensations experienced during the significant event implicated in its etiology (11).

Given the central role of mental imagery in the maintenance of a variety of mood and anxiety disorders, cognitive therapy researchers have developed novel interventions aimed at promoting less threatening appraisals of recurrent imagery through “rescripting” (12–14). Wheatley and Hackmann (15) suggest that imagery rescripting may lead to reappraisals of negative or traumatic experiences, resulting in fundamental shifts in current cognitive processing in a similar manner to that observed following the use of verbal cognitive therapy strategies. These previous studies of imagery rescripting, especially in anxiety disorders, encouraged us to develop and test an imagery rescripting procedure for BBCA. Two observations, in particular, support the use of such an approach in this population. First, as noted above, there is a high level of reported intrusive mental imagery related to loss of bowel/bladder-control in this group (7). More importantly, however, our experience of treating patients with BBCA suggests that it is often critical to acknowledge the importance of, and develop a treatment focus around past experiences characterized by shame and lack of autonomy or control. Although these broad themes are amenable to verbal cognitive strategies, it seems more parsimonious to match treatment strategies to the phenomenology of the symptoms in question: that is, to use imagery rescripting to tackle imagery because the latter seems to predominate as a cognitive symptom in BBCA.

## Method

### Design

A case series design was used (16) with assessments at three time points: immediately pre- and post-imagery rescripting and 1-week after the session (“follow-up”). Longer-term follow-up was not appropriate for a single-session experimental study. The sessions were conducted by a researcher-therapist who was a Graduate Student in Clinical Psychology (Rosanna Pajak), and supervised by a Research Clinical Psychologist accredited in Cognitive Therapy (Sunjeev K. Kamboj).

The study was approved by University College London/University College London Hospital Research Ethics Committee.

### Procedure

Sessions (~1.5 h) were conducted in a quiet room in an academic clinical psychology department. The session began with a diagnostic interview, which aimed to identify the presence of anxiety disorders (see below).

A rationale for rescripting was provided, which was designed to clarify the aims of the intervention as an experimental procedure. On the basis of this description, participants rated the procedure’s credibility (17). Participants were then asked to identify and imaginatively elaborate a “safe place” in case of intense distress. On the basis of a semi-structured interview (8), the researcher-therapist elicited distressing mental imagery associated with the participant’s experience of BBCA. When more than one distressing image was reported, participants were asked to select the image that caused most distress/impairment in daily life. Participants were encouraged to allow the emotional response to the image to be fully experienced during the session, although strategies to regulate emotions were also discussed (e.g., opening eyes or switching to the alternative, “safe place” image).

As described by Wheatley and Hackmann (15), participants recalled their primary distressing image, described it in detail, and then rating it on a number of dimensions (see Measures below). With this distressing image held in mind, participants were asked: “What needs to happen in this scene for you to feel okay?” as a way to initiate image transformation. Given the hypothesized presence of high levels of shame and beliefs about loss of control, participants were encouraged to evoke imagery transformations that included a sense of compassion and mastery. As image transformation unfolded, qualitative descriptions of changes in content and emotional response were elicited. After rescripting, participants re-rated their image, followed by a “grounding” procedure.

Before leaving the session, participants were asked to practice replacing distressing BBCA-related imagery with the rescripted image throughout the following week. A follow-up telephone interview elicited additional imagery ratings at 1-week. All participants received a small reimbursement for their time and travel expenses.

### Participants

Three participants were recruited from a pool (*n* = 23) of those who had previously taken part in a research project exploring the characteristics of mental imagery in BBCA. An inclusion criterion for the current study was the absence of any organic condition that might be associated with frequent incontinence (e.g., an organic bowel disease). In addition, participants needed to identify a memory of a distressing past event that was linked to the experience of current BBCA-imagery (i.e., a “flashback”) (8). Of the nine participants who met these criteria, six declined/could not be contacted.

### Participant 1

This participant was a 26-year-old white-British man completing postgraduate studies and reporting bladder-control anxiety (fear of urinary incontinence) since age 16. The onset was after a “near miss” on a school trip. He reported one to three episodes of intense anxiety related to fears of losing bladder-control each week. He had neither sought professional help nor told any friends or family members about the nature of his anxiety. He met diagnostic criteria for agoraphobia without panic disorder (see Measures below).

Participant 1’s imagery content varied according to the situation (e.g., queuing for the bathroom, visiting an unknown shopping mall) but generally involved future-oriented images of searching for somewhere to “relieve” himself. He linked this imagery to the distressing experience of the “near miss” aged 16, the central narrative of which was transposed onto different, contemporary situations.

### Participant 2

The participant was a 61-year-old white-British man in full-time employment reporting bladder-control anxiety. He described himself as “self-conscious and depressed” throughout childhood and adolescence, which he related to an experience of urinary incontinence at school, aged 6. His confidence improved in early adulthood, but symptoms of BBCA, paruresis, and depression re-emerged at age 40. He experienced two to three episodes of anxiety related to bladder-control each *day* (e.g., in meetings at work, or in social settings like the cinema or theater). The content of his imagery often related to his experience at age 6. He met diagnostic criteria for social phobia.

### Participant 3

This was a 31-year-old white-British woman in full-time employment reporting bowel-control anxiety since age 24 and experiencing future-oriented imagery when using public transport (e.g., being trapped in a crowded train carriage and experiencing intense gastrointestinal sensations and panic). She related this imagery to a “near miss” while suffering from gastroenteritis on holiday, aged 24. She explained that she had not felt able to think about this experience at all for many years afterward. Like Participant 1, the central narrative appeared to be transposed onto contemporary experiences. Participant 3 had received a diagnosis of irritable bowel syndrome (IBS) and previously sought help from a hypnotherapist. She believed her IBS/gastrointestinal symptoms were “stress-related” and described anxiety about “flare-ups,” causing bowel-related anxiety almost every day. She met diagnostic criteria for agoraphobia without panic disorder.

### Measures

#### Credibility assessment

We used three credibility items assessing logic of the “treatment” approach, expectations regarding usefulness and confidence in recommending the “treatment” from the credibility/expectancy questionnaire (CEQ) (17). Ratings are from 1 (not at all logical/useful/confident) to 9 (very logical/useful/confident) with possible scores ranging from 3 to 27. An expectancy item from the CEQ relating to expected symptom improvement was also used (rated 0–100%).

#### Diagnostic assessment

Clinical diagnoses were evaluated using the structured clinical interview for DSM disorders (SCID) anxiety module (18).

#### Imagery ratings

After describing the content of imagery participants rated image distress, vividness, and “nowness,” experience of various emotions (anxiety, shame, and disgust) and intensity of physical sensations while holding the image in their mind were also rated on a 0–100 scale. After identifying the worst aspect of their image, its meaning to “the self,” other people and/or their body was elicited in the form of an encapsulated belief, which was rated for strength of conviction on a 0–100 scale. After rescripting, participants recalled their original image and re-rated it using the same measures. These imagery ratings were obtained again at 1-week.

## Results

### Procedure credibility

Total CEQ credibility (based on the sum of the three credibility items) was lower for Participant 2 (13/27) than Participants 1 (20/27) and 3 (22/37). Improvement expectancy was 70% (Participant 1), 30% (Participant 2), and 20% (Participant 3).

### Response to imagery rescripting

#### Participant 1

Participant 1’s imagery involved asking for the school bus to be stopped so he could use a bathroom. Accompanying the imagery are feelings of being self-conscious and fearful that other students had become aware of his desperate need to use the bathroom. He described a sense of being “really trapped,” and of trying to control both his panic and need to urinate while reassuring himself that the bus will stop soon. The memory then jumps forward to an image of reaching the bathroom but seeing a female classmate enter before him. He then described “agony” in his bladder, restlessness, and extreme physical and mental tension. Upon entering the bathroom, he saw that he has splashed some urine on himself leading to concern that this “accident” will be discovered by his classmates.

**Figure 1:**
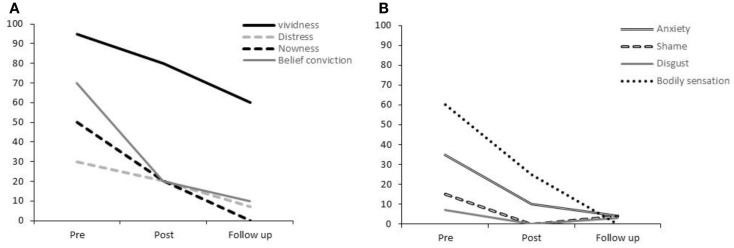
**Participant 1’s ratings for (A) vividness, distress, nowness, and strength of belief conviction and (B) anxiety, shame, disgust, and bodily sensations**.

Prior to rescripting, Participant 1 experienced this image as highly vivid (95%), although the associated distress was not high (30%; see Figure 1A). The belief accompanying this imagery was summarized thus: “I am not in control of my own body, or even my own mind,” which was held with a moderate-high level of conviction (70%). Anxiety was the strongest emotion associated with the image, rated at 35%. It was noted that Participant 1 had not experienced the image at all in the week before the rescripting session.

When rescripting this image, Participant 1 said that he needed “to feel more in control.” He would have liked to have seen himself as having more confidence and control in the situation. When encouraged to explore what might need to change in his image to provide him with confidence, he envisaged his partner sitting beside him on the school bus, expressing understanding of his distress and calmly and gently informing him that there is “no harm in asking for the bus to stop.” Participant 1 reflected that he needed to hear that “it is not unusual to have to ask to use the bathroom: this happens to lots of people.” Introducing this normalization and reassurance into the image enabled him to feel more relaxed and confident. When focusing on the feeling of confidence in the image, he described seeing a “brighter day outside the bus windows” and feeling his shoulders and chest rising in confidence. He then imagined himself confidently explaining his need to use the bathroom to the bus driver and the bus driver responding with compassion, providing reassurance that a bathroom is nearby and telling him that stopping is not a problem. In addition, he imagined fewer people on the bus, giving him the sense that he was not inconveniencing many other students. Participant 1 then reported that he then felt “relaxed, calm, empowered, and in control.” He explained that the physical sensations of a full bladder (in the image) had reduced to a manageable level, without pain or urgency. Finally, he imagined himself reaching and using the bathroom in this calm state.

As shown in Figure 1A, immediately after this rescripting, the vividness of Participant 1’s original image had reduced by 15% and the associated distress by 30%. Shame and disgust were at relatively low-levels prior to rescripting but were rated at 0% following rescripting (Figure 1B). Anxiety, bodily sensations, “nowness,” and belief conviction ratings all reduced by more than 50%.

One-week later, Participant 1 reported further reductions in vividness, distress, anxiety, and the strength of belief. He has no longer experienced bodily sensations while holding the image in mind or a sense of current re-experiencing (“nowness”). He explained that he could picture the original image but felt in control of his body, possibly due to the absence of physical sensations. He reported the occurrence of one episode of bladder-control related anxiety during the week, to which he responded by recalling the rescripted image.

#### Participant 2

Participant 2’s memory was of a distressing experience of urinary incontinence aged 6. He recalled losing control of his bladder in a school assembly, this being discovered and the negative response of his teacher. During imaginal reliving, he imagined being seated on the floor and experiencing unpleasant sensations of needing to urinate. He imagined himself as restless, hot, anxious, and becoming increasingly distressed. He recalled his reasoning process at the time: since he often found it difficult to use the school bathroom, he considered it to be preferable to stay seated and regarded it as unlikely that others would notice if he wet himself. He then described the sensation of his stomach dropping in fear as he “decided to let go” followed by a brief sense of physical relief, until the end of the class when he had to stand up at. At that point, he and others realized that there was a large puddle on the floor. He described feeling extremely distressed, helpless, and vulnerable. He recalled the teacher sternly asking who was responsible and then desperately denying that it was him. He recalled the teacher saying “You’re dirty: go to the toilet in the proper place!” and sending him out of the room, leaving him feeling distressed and ashamed. Sobbing, he made his way to the bathroom. He also recalled another teacher impatiently asking “What’s the matter now?” and repeating that he should have used the bathroom.

**Figure 2:**
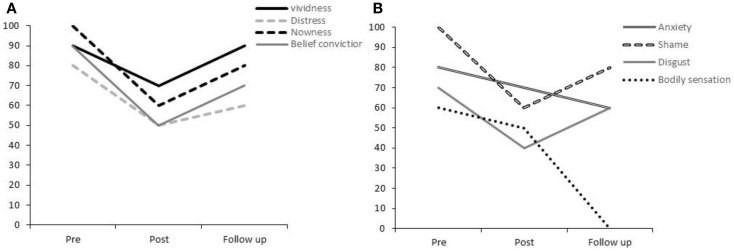
**Participant 2’s ratings for (A) vividness, distress, nowness, and strength of belief conviction and (B) anxiety, shame, disgust, and bodily sensations**.

Prior to rescripting, Participant 2 experienced this image as highly vivid (90%) and distressing (80%). When exploring the image-associated belief, he explained that he had taken the reactions of the teachers to mean “I am bad, I am naughty.” He reported a high level of belief conviction (90%). The emotion most strongly associated with the image was shame (100%), although anxiety and disgust were also high (>70%; Figure 2B). Participant 2 expressed the addition thought “I should not have let this happen.”

During rescripting, a number of themes emerged for Participant 2. Initially, he expressed a strong desire for somebody to “rescue” him: to take charge of the situation, respond compassionately, and support him to leave the situation discreetly. He imagined a particular friend as his rescuer, someone who had previously responded compassionately when told about this event. Participant 2 envisaged this friend reassuring him, taking him by the hand and walking with him out of the room. He described a physical sense of comfort and relief but continued to experience self-blame. He expressed a desire for his friend to hug him, but was concerned that his friend would not want to get his clothes wet. The researcher-therapist suggested imagining that the friend was not concerned about this and Participant 2 was subsequently able to imagine being held and feeling warm and safe. As he continued to feel a sense of responsibility and self-blame, Participant 2 chose to imagine being able to explain his dilemma: that he found it difficult to use the school bathroom and therefore felt he had no other options. He imagined an understanding, normalizing, and reassuring response from his friend, particularly receiving the strong message that Participant 2 was not alone – others have had the same experience. Participant 2 also wanted to imagine his friend helping him to change into a clean, dry school uniform, and accompanying him to help explain the reasons behind the accident to his mother, thus, reducing anxiety about being reprimanded again. At this stage, Participant 2 visualized the two of them entering a quiet room and his friend telling him that there is nothing wrong with him for not being able to use the school bathroom, and that most men and boys experience this difficulty. At this point, he experienced a sense of “a great weight being lifted” and intense warmth between himself and his friend.

During the rescripting, Participant 2 described feeling “reassured” but continued to feel that there is some “stigma” attached to his experience. He related this to concern about the opinions of other children. Participant 2 chose to imagine that his teacher had told the class not to mention it, although this did not appear to reduce his sense of being “inferior” to others. He therefore chose to give his “child-self” the knowledge that people are not “appalled” by incontinence – that it is not such a “monumental” occurrence and he is not a bad person as a result of the experience. He described feeling significantly less anxious and ashamed after this aspect of the image transformation. His final remaining concern was that the floor would still be wet the following day, so he imagined walking with his friend to see the floor and feeling reassured that nothing could be seen.

Immediately, after rescripting, Participant 2’s ratings for his original image were reduced across all domains by between 20 and 40% (see Figures 2A,B). At 1-week follow-up, his imagery ratings had generally increased from the post-rescripting time-point, although not reaching the high level of his original ratings. Notably, there was an absence of physical sensations associated with the image, which he rated at 0%. He reported that the rescripted image seemed to spontaneously come to mind whenever he had experienced the original image during the week. He had experienced the occurrence of this competing image as reducing the emotional impact of the original memory.

#### Participant 3

Participant 3 described a primary distressing image of a “near miss” during a driving holiday with her parents aged 24. She explained that she had been ill with gastroenteritis but had felt reluctant to tell her parents that she did not feel well enough to embark on a road trip. During imaginal reliving, she imagined herself in the rear seat; experiencing gastrointestinal sensations of pain and tension on the left side of her abdomen: “inflamed and sore … like a steel case.” She recalled increasing feelings of panic, tension, and distress as she searched the roadside for a convenient place to stop. She described a sense of intense shame at not being able to make it through the journey without stopping and anxiety about asking her father to stop, as she anticipated an angry reaction from him. She reported a fear that she would lose control of her bowels and be “humiliated.” She described anger, disappointment, and frustration toward her father for holding all of the control over the journey. She also vividly recalled his angry response when she did ask him to stop, and his initial refusal to pull over. Once a bathroom was found, she experienced feelings of embarrassment and guilt for delaying their journey.

The beliefs associated with her image were summarized by thus: “I am childish for needing the toilet and because I have no control over this situation; my needs and choices are not being respected.” It was noted that the physical sensations of pain were particularly vivid within her image.

During rescripting, Participant 3 expressed her need for her father to respond with compassion, but found this too difficult to imagine. Instead, her rescripted image involved making a more assertive, forceful request to stop the car earlier in the journey, before the urge had become so intense. She also envisaged resting her hand on the left side of her abdomen and imagined a cool, “Aloe Vera type” balm flowing into her gut, reducing inflammation and soothing her pain. She subsequently described feeling more in control. In her rescripted image, she visualized seeing a clean set of bathrooms from the car window, with no queue and a safe place to park. She then imagined a small shop beside the bathrooms and had her father spontaneously decide that they should stop there, which removed her feelings of guilt and embarrassment. At this point, Participant 3 described feeling relaxed and noted that her previous sense of feeling childlike had diminished. Lastly, she introduced a compassionate response from her mother and her friend, imagining them responding to her distress with reassurance that she was not inconveniencing them, and normalizing her experience by saying “it’s completely understandable, happens to everybody.” She then described feeling comforted and understood.

**Figure 3:**
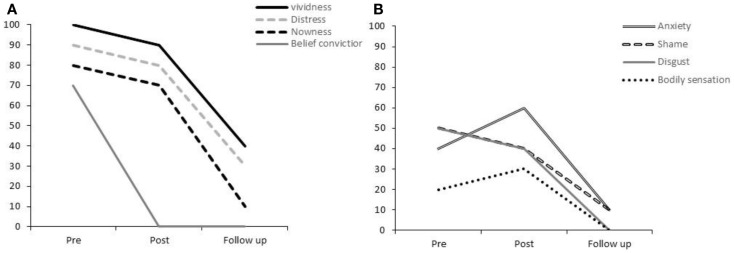
**Participant 3’s ratings for (A) vividness, distress, nowness, and strength of belief conviction and (B) anxiety, shame, disgust, and bodily sensations**.

Immediately, after this rescripting, Participant 3 reported slight (10–20%) reductions in vividness, “nowness,” distress shame, and disgust (Figure 3), while reporting 30% increases in the intensity of the bodily sensations and anxiety associated with her original image. Of particular note, she reported an immediate and total reduction of her encapsulated belief following rescripting.

At 1-week follow-up, Participant 3 reported significant reductions in the impact of her BBCA-imagery. Her scores for vividness, distress, “nowness,” anxiety, and shame had almost reduced to 0% (see Figures 3A,B). The belief conviction rating of 0 had been maintained from the post-rescripting time-point. As with Participant 2, there was a complete absence of physical sensations while the image was held in mind.

She explained that she had continued to “play around” with different rescripted images in her mind the day after the session. She stated that both discussing the image in detail and changing it in her imagination appeared to have significantly reduced its emotional impact. She noted that with her more adult perspective, she was even able to reflect on the experience as slightly humorous.

## Discussion

We describe preliminary outcomes of a case series exploring the value of a single-session imagery rescripting session in BBCA, a viscerally centered phobic syndrome. Similar brief imagery-based interventions have been successfully implemented for social anxiety (19, 20), depression (21), and specific phobia (12). Our findings suggest that the efficacy of rescripting may extend to this neglected syndrome, for which there are currently no published treatment protocols.

Across the three participants, reductions were noted in distress, “nowness,” anxiety, shame, and strength of belief associated with the distressing memory. For Participants 1 and 3, reductions of at least 50% were observed, either after 1-week (Participant 3) or immediately after rescripting and maintained at 1-week follow-up (Participant 1).

While all three participants seemed to benefit from the rescripting procedure, Participant 2’s overall response was smaller. However, his pre-treatment levels of shame, disgust, and anxiety were higher. Moreover, he rated the treatment as less credible than the other two participants. Finally, his difficulties were more chronic (>50 years). This may simply suggest that those with chronic symptoms require a more consolidated treatment approach and preparatory work aimed at improving treatment acceptability.

All three participants reported a complete abolition of image-associated bodily sensations at follow-up. All participants experienced their memories “as though they were happening again right now” (“nowness”) during imaginal reliving, with the physical sensations being particularly intensely. The lack of activation of these sensations upon memory retrieval at 1-week follow-up is therefore especially striking.

Beliefs about loss of bodily control and the presence of strong physical sensations could contribute to “visceral sensitization” via positive feedback loops forming the gut–brain axis (7). The finding that imagery rescripting significantly reduced the occurrence of physical-sensory memories in all three participants suggests that this is a potentially useful treatment strategy for those who experience sudden urgency in anxiety-provoking situations. This could represent a helpful preparation for graded exposure and may also be helpful in other disorders characterized by gastrointestinal urgency, such as IBS.

Imagery in BBCA tends to be characterized by helplessness and shame (8). Alternatively, and in line with clinical intuition, the rescripted images highlight the importance of control and mastery in BBCA. Given that reductions were noted in the participants’ level of belief in their original cognition, as well as the associated emotional experiences of shame and anxiety, it appears that creative memory rescripting can lead to new appraisals of distressing past experiences in people with BBCA.

It is noteworthy that all three participants benefited from replacing the original intrusive memory with the rescripted image when it occurred during the 1-week follow-up period. This is in line with the idea that imagery rescripting creates an alternative representation of the distressing memory that will not only give new meaning to the experience but also competes with the original memory representation when it is triggered (22).

The usual limitations associated with case series descriptions apply to this study (16). However, at this early stage of treatment-development for this distressing and neglected syndrome, our purpose was to demonstrate a proof-of-principle rather than generalizability. Thus, while it is acknowledged that the follow-up period was brief, our aim was not to show enduring effects, but rather the ability of rescripting to produce acute effects, which if repeated over multiple sessions, may produce longer-term beneficial effects.

This first study of imagery rescripting in people with BBCA suggests that this is a promising approach to treating a neglected syndrome for which there are no specific current specific treatments.

## Conflict of Interest Statement

The authors declare that the research was conducted in the absence of any commercial or financial relationships that could be construed as a potential conflict of interest.
